# Higher Incidence of Venous Thromboembolism in Anterolateral Approach in Lateral Position Compared to Anterolateral Supine and Direct Anterior Approaches in Minimally Invasive Total Hip Arthroplasty

**DOI:** 10.7759/cureus.66831

**Published:** 2024-08-14

**Authors:** Toru Nishiwaki, Akihito Oya, Atsuhiro Fujie, Arihiko Kanaji

**Affiliations:** 1 Deaprtment of Orthopedic Surgery, Japanese Red Cross Shizuoka Hospital, Shizuoka, JPN; 2 Department of Orthopedic Surgery, Keio University School of Medicine, Tokyo, JPN; 3 Department of Orthopedic Surgery, Fujita Health University Bantane Hospital, Nagoya, JPN

**Keywords:** an anterolateral approach, a direct anterior approach, venous thromboembolism, total hip replacement, minimally invasive surgery

## Abstract

Introduction: Venous thromboembolism (VTE) remains a major complication after total hip arthroplasty (THA), irrespective of the surgical approach. This study investigated the incidence of VTE in patients undergoing THA through intermuscular minimally invasive surgical techniques, which included a direct anterior approach (DAA), an anterolateral approach (AL), and an anterolateral supine approach (ALS), at a single institution.

Methods: A hundred consecutive patients treated with each surgical approach were evaluated. Plasma D-dimer levels one month preoperatively and one day postoperatively, operative time, and intraoperative blood loss were recorded, and the presence of VTE was evaluated based on multidetector-row computed tomography performed the day after surgery. Student’s t-test and Pearson’s chi-square test or one-way analysis of variance were used in statistical analysis.

Results: No differences among the groups in terms of age, height, weight, operative time, intraoperative bleeding, and preoperative and postoperative D-dimer levels were observed. The overall incidence of VTE was 21%. The incidences of VTE were 30% in AL, 17% in ALS, and 16% in DAA, representing a significantly higher rate in AL than in ALS and DAA (P=0.025). The incidences of VTE on the operated side were 19% in AL, 13% in ALS, and 12% in DAA, with no statistically significant differences. The incidences of VTE on the non-operated side were 22% in AL, 9% in ALS, and 8% in DAA; these differences were statistically significant (P=0.0045).

Discussion: Results showed that the incidence of VTE was significantly higher in AL than in ALS and DAA, especially for the non-operated side.

## Introduction

Total hip arthroplasty (THA) has been considered one of the most successful operations in orthopedic surgery for over 40 years. However, for the past decade, considerable interest has been devoted to the development of minimally invasive surgical techniques (MISs). MISs include the following: a direct anterior approach (DAA), an anterolateral approach (AL), and an anterolateral supine approach (ALS). DAA involves accessing the hip joint between the tensor fascia lata and the sartorius muscles, leveraging the inter-nervous plane between the femoral and superior gluteal nerves. Additionally, the modified Watson-Jones anterolateral approach has also been used as a minimally invasive approach for THA, working through the interval between the tensor facia lata and the gluteus medius. The approach can be performed in both the supine (ALS) and lateral (AL) positions. MISs for THA have now become popular worldwide. The advantages of MISs include less soft tissue trauma (smaller skin incisions and less muscle damage), reduced blood loss, and fewer blood transfusion requirements. Postoperative benefits include less pain, shorter hospital stays, quicker return to function, and a better cosmetic appearance [1-3].

In terms of the postoperative complications after THA, dislocation, one of the most common causes of revision hip surgery, has decreased with using these anteriorly based surgical approaches. However, venous thromboembolism (VTE) remains a major complication in patients after THA, irrespective of the surgical approach [4].

Despite the prevalence of various minimally invasive surgical techniques, there is still a lack of detailed reports on the incidence of VTE for each approach. Moreover, it is often observed that VTE can occur in both the operated and non-operated limbs following THA, yet studies comparing the incidence between the operated and non-operated limbs are also insufficient. The purpose of this study is to elucidate the overall incidence of VTE for each approach, compare the incidence of VTE between the operated and non-operated limbs, and determine if there are any statistically significant differences.

## Materials and methods

This retrospective study was approved by the ethics committee (Keio University Ethics Review Board, approval no. 2015001) of our facility, and informed consent was waived because of the retrospective nature of this study. This article was previously posted to the medRxiv preprint server on April 27, 2020.

Patients were included if they were aged 50 to 80 years, female, had a body mass index (BMI) <30 kg/m2, were diagnosed as having mild osteoarthritis of the hip (up to Crowe type 2) due to developmental dysplasia of the hip, and had no prior history of hip surgery (e.g., pelvic osteotomy). In our hospital, between April 2007 and January 2018, three experienced surgeons were assigned to use three different minimally invasive intermuscular approaches, including AL, ALS, and DAA. Each surgeon had performed THA using an MIS as an intermuscular approach in over 500 cases before working at our hospital. A hundred consecutive patients treated with each approach, beginning seven months after each experienced surgeon was assigned to our hospital, were evaluated. Surgeons skilled in each approach considered patients seen in their own outpatient clinics as candidates for their approach. In all patients with normal renal function, without known allergies to contrast agents, and who provided consent, contrast multidetector row computed tomography (MDCT) was performed to examine the presence of VTE on the day after surgery. Patients who underwent simultaneous bilateral THA had a history of VTE, or received anticoagulants preoperatively were excluded. All implants were non-cemented prostheses, and all operations were performed under general anesthesia with spinal regional anesthesia.

The plasma D-dimer levels one month preoperatively and one day postoperatively, operative time and intraoperative blood loss were recorded, and the presence of VTE was evaluated based on MDCT performed the day after surgery and read by a radiology specialist at our university.

Thromboprophylaxis

Mechanical thromboprophylaxis, including pressure stockings and compression devices, was started immediately on the operated side postoperatively and on the non-operated side intraoperatively. Patients who were not diagnosed as having renal or hepatic failure received chemoprophylaxis therapy after MDCT on the day after surgery. Chemoprophylaxis therapy included enoxaparin, edoxaban, danaparoid, or fondaparinux. Patients were allowed to be fully weight-bearing after confirming the absence of VTE involving the proximal vein.

Statistical analysis

The Student’s t-test was used to analyze parametric data. Patient background factors were examined between groups using Pearson’s chi-square test or one-way analysis of variance, and P < 0.05 was considered statistically significant. IBM SPSS Statistics for Windows, Version 25 (IBM Corp., Armonk, NY) was used to perform statistical analyses in this study.

## Results

No differences between groups in terms of age, height, or weight were observed with respect to patient background characteristics (Table 1).

**Table 1:** Patient demographic characteristics. Data are shown as mean ± standard deviation. AL, anterolateral approach; ALS, anterolateral supine approach; DAA, direct anterior approach; N.S.: not significant.

	AL	ALS	DAA	P-value
Age (years)	66 ± 8	67 ± 8	68 ± 8	N.S.
Height (cm)	153 ± 5	154 ± 5	150 ± 6	N.S.
Weight (kg)	54 ± 10	53 ± 9	56 ± 9	N.S.
Body mass index (kg/m^2^)	23 ± 4	22 ± 4	26 ± 4	N.S.

The operative times in each group were as follows: 96 ± 23 minutes in AL, 100 ± 20 minutes in ALS, and 94 ± 24 minutes in DAA. The amounts of intraoperative blood loss were 188 ± 102 ml in AL, 215 ± 111 ml in ALS, and 220 ± 156 ml in DAA. These differences were not statistically significant (Table 2). The mean preoperative D-dimer values in each group were as follows: 0.81 ± 0.73 g/ml in AL, 1.10 ± 1.07 g/ml in ALS, and 0.75 ± 0.80 g/ml in DAA. The postoperative day 1 D-dimer values were 12.8 ± 10.5 g/ml in AL, 11.8 ± 10.7 g/ml in ALS, and 13.2 ± 11.4 g/ml in DAA. No statistically significant differences were identified.

**Table 2:** Perioperative results. Data are shown as mean ± standard deviation. AL, anterolateral approach; ALS, anterolateral supine approach; DAA, direct anterior approach; N.S.: not significant.

	AL	ALS	DAA	P-value
Operative time (min)	96 ± 23	100 ± 20	94 ± 24	N.S.
Intraoperative blood loss (ml)	188 ± 102	215 ± 111	220 ± 156	N.S.
Preoperative D-dimer level (g/ml)	0.81 ± 0.73	1.10 ± 1.07	0.75 ± 0.80	N.S.
Postoperative D-dimer level (g/ml)	12.8 ± 10.5	11.8 ± 10.7	13.2 ± 11.4	N.S.

**Figure 1:**
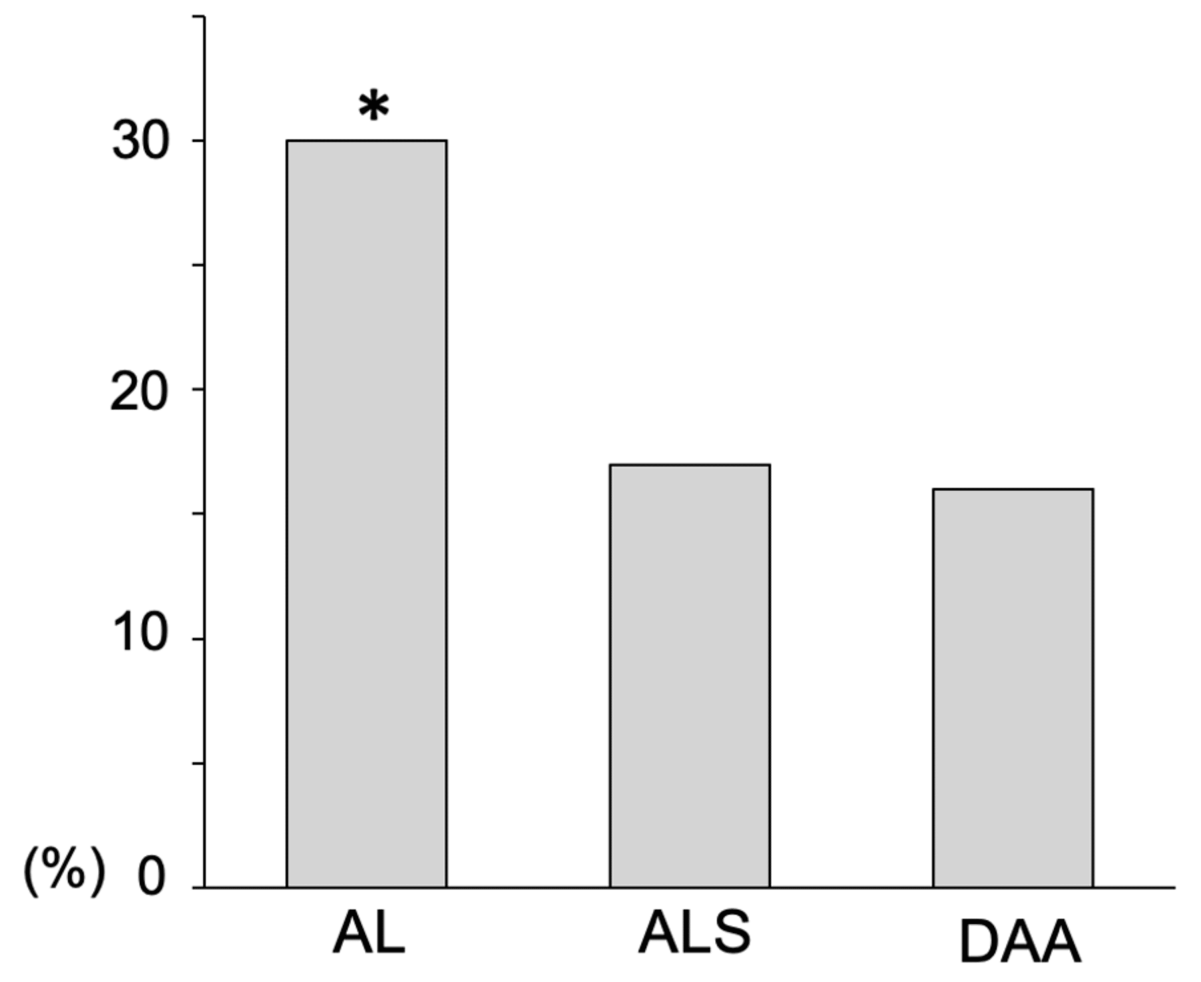
The overall incidence of VTE. The incidences of venous thromboembolism (VTE) are 30% in the anterolateral approach (AL), 17% in the anterolateral supine approach (ALS), and 16% in the direct anterior approach (DAA). The incidence of VTE is significantly higher in AL than in ALS and DAA (P=0.025). * P < 0.05.

The overall incidence of VTE was 21% (63/300). The incidences of VTE were 30% (30 of 100) in AL, 17% (17 of 100) in ALS, and 16% (16 of 100) in DAA (Figure 1).

**Figure 2:**
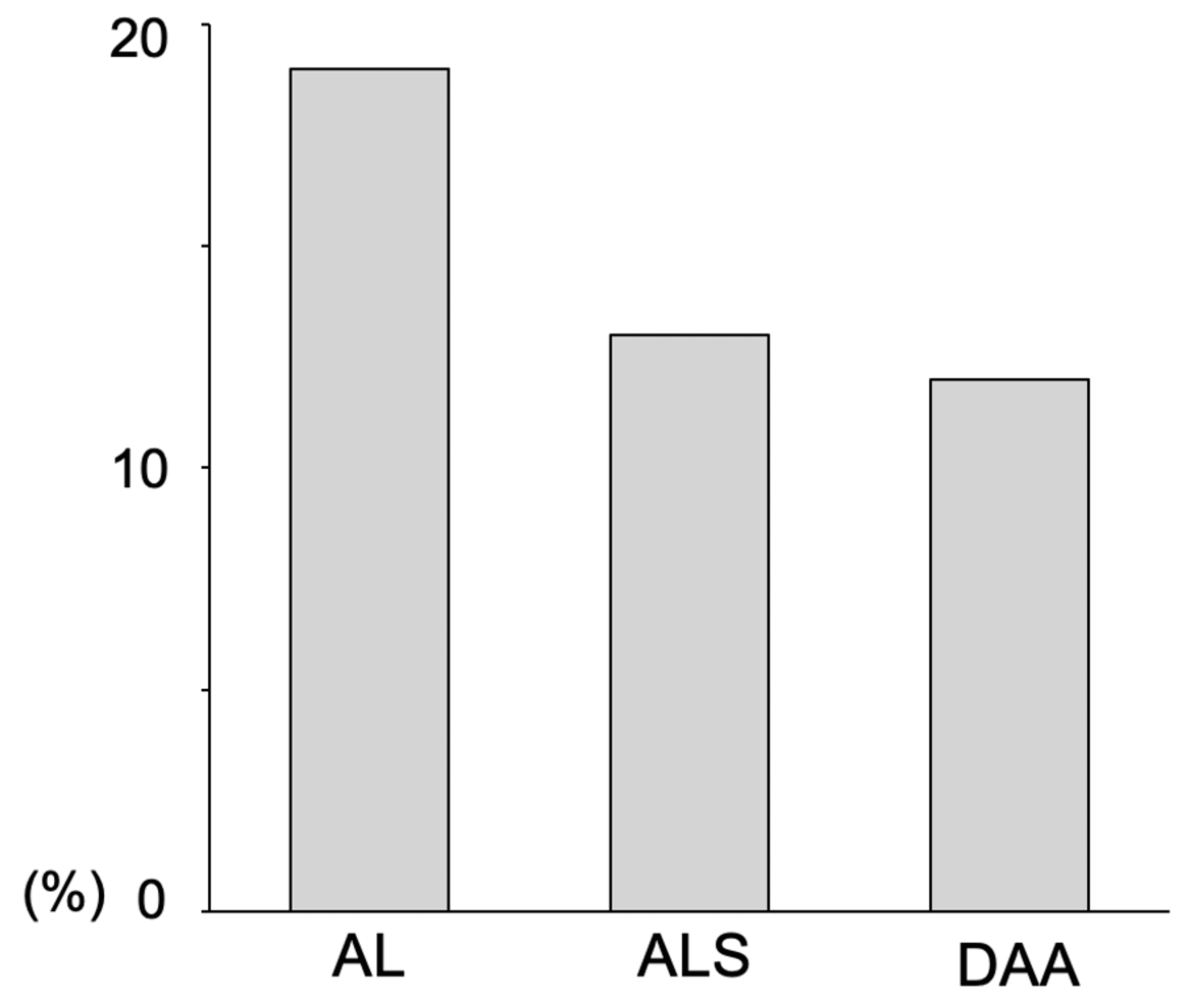
The incidence of VTE on the operated side. The incidences of venous thromboembolism (VTE) on the operated side are 19% in the anterolateral approach (AL), 13% in the anterolateral supine approach (ALS), and 12% in the direct anterior approach (DAA) (P=0.32).

The incidence of VTE was significantly higher in AL than in ALS and DAA (P = 0.025). The incidences of VTE on the operated side were 19% in AL, 13% in ALS, and 12% in DAA; these differences were not statistically significant (P = 0.32) (Figure 2).

**Figure 3:**
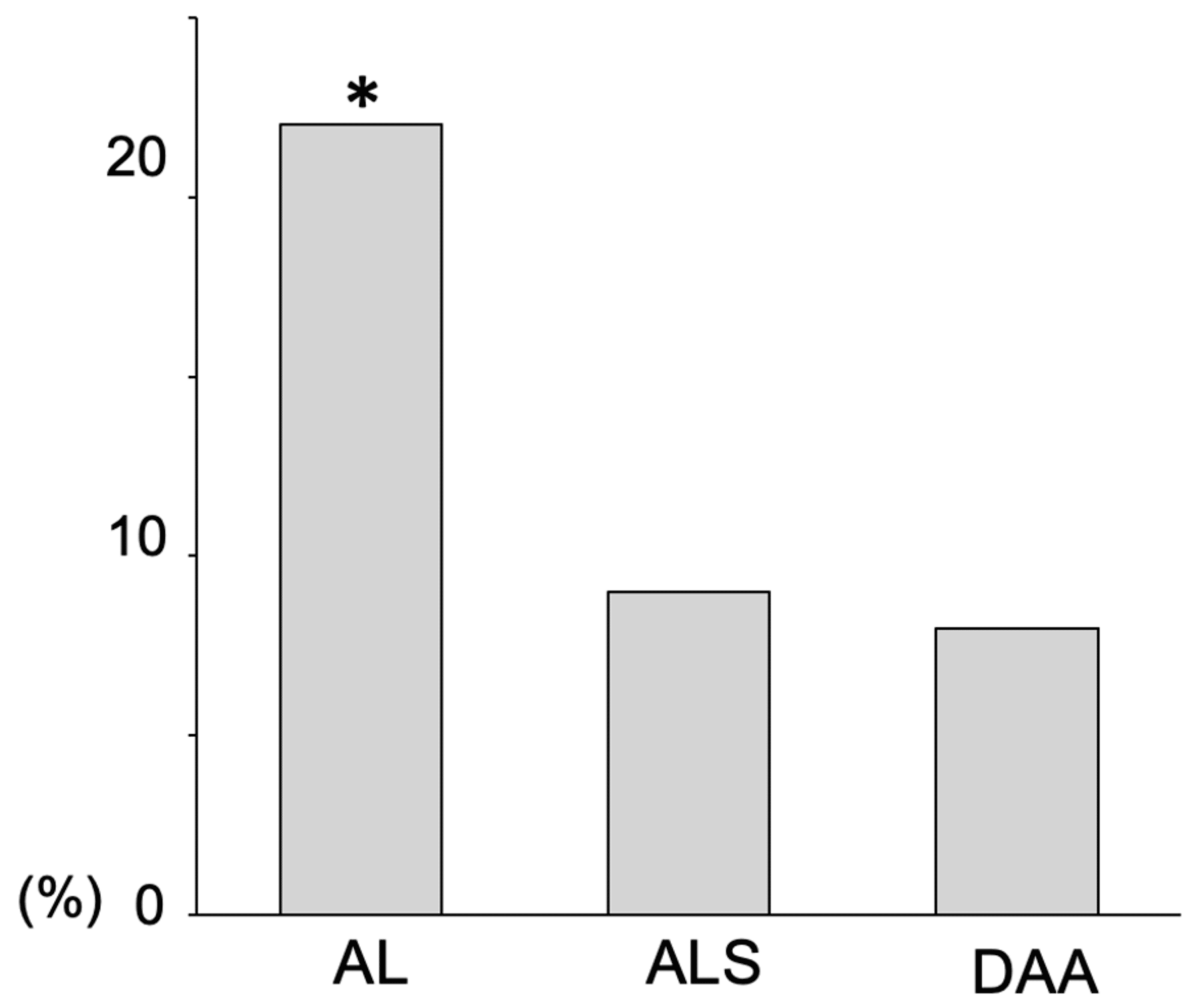
The incidence of VTE on the non-operated side. The incidences of venous thromboembolism (VTE) on the non-operated side are 22% in the anterolateral approach (AL), 9% in the anterolateral supine approach (ALS), and 8% in the direct anterior approach (DAA). The incidence of VTE on the non-operated side is significantly higher in AL than in ALS and DAA (P=0.0045). * P < 0.05.

The incidences of VTE on the non-operated side were 22% in AL, 9% in ALS, and 8% in DAA; these differences were statistically significant (P = 0.0045) (Figure 3).

The incidences of VTE involving the proximal vein were 2% in AL, 2% in ALS, and 2% in DAA. The incidences of pulmonary embolism were 6% in AL, 3% in ALS, and 6% in DAA. All patients were asymptomatic.

## Discussion

The anterior intermuscular approaches, which include AL, ALS, and DAA, allow for implantation without damage to muscles, and they were developed to reduce postoperative bleeding, accelerate patient recovery, and improve early clinical results. The potential effect of early recovery can be expected to reduce the incidence of VTE. In this study, the incidence of VTE was evaluated before ambulation and chemoprophylaxis. The present study is, to our knowledge, the largest reported series of THA at a single institution comparing different minimally invasive intermuscular approaches that focuses on the incidence of VTE. We found that the incidence of VTE in AL was significantly higher than that in ALS and DAA.

VTE is believed to be associated with local vessel damage, decreased venous flow, and surgical hypercoagulation. Previous studies of venous blood flow during THA using a standard posterior approach demonstrated that femoral vein occlusion occurred with flexion, adduction, and internal rotation (the position of posterior dislocation) and with placement of retractors around the acetabular rim [5,6]. Some studies showed that external rotation and extension, the positioning used for acetabular and femoral preparation for the anterior intermuscular approach, had no effect on femoral vein flow, as the femoral vein moves anterolaterally, which protects against compression between the proximal femur and pubic bone [5,7]. Therefore, we hypothesized that the femoral vein in THA performed via the anterior intermuscular approach, including AL, ALS, and DAA, would be at a low risk for occlusive events that could increase the risk for thrombosis.

We found that the incidence of VTE in ALS and DAA is comparable, and that in AL, especially on the non-operated side, the incidence of VTE was significantly higher than that in ALS and DAA. Patients were positioned in the supine position in ALS and DAA, whereas they were positioned in the lateral position in AL. Postural changes in venous diameter due to gravity have been reported; in other words, the diameters of the right femoral vein in the right lateral position and left femoral vein in the left lateral position were reported to be significantly larger than those in the supine position [8]. Hence, there is a possibility that the femoral venous flow of the non-operated side in the lateral position may be slower than that in the supine position, resulting in a higher incidence of VTE.

There is a report that the right femoral vein may be at higher risk for VTE in the right lateral position than in the supine position [8]. According to this report, there are two possible causes. First, the inferior vena cava may be compressed by abdominal viscera in the right lateral position, resulting in decreased blood return from the lower extremities. Second, sympathetic tone is lower in the right decubitus position [9], and peripheral venous dilatation may occur, which may decrease venous return and velocity. However, in the current study, a difference in the incidence of VTE between the right leg in the right decubitus position (11%: 5 of 44) and the left leg in the left decubitus position (11%: 6 of 56) was not seen in AL.

On the operated side, there was a tendency toward an increased incidence of VTE in AL compared to that in ALS and in DAA. The leg position itself is almost equivalent among these approaches, and it has been reported to be safe for femoral venous blood flow in 100% of patients during acetabular exposure and 80% of patients during femoral exposure [7]. We speculate that decreased blood return from the lower leg, due to lowering the operated leg toward the floor in AL, may be a cause of the higher VTE incidence [10] compared to that in the supine position.

The placement of an anterior single-pronged Homan-like retractor over the anterior wall during acetabular preparation in the anterior approach is reported to be a cause of consistent occlusion of the femoral vein [7], whereas positioning the leg for acetabular and femoral preparation did not lead to occlusion of the femoral vein. In the present study, we did not measure retraction time. However, the operative time was not significantly different between the groups, and we believe the period of retractor placement was thus not significantly different between the approaches.

Our study has several limitations. First, our results are representative of only 50 to 80-year-old, Japanese, female patients with a BMI <30 kg/m2 diagnosed as having mild osteoarthritis of the hip (up to Crowe type 2). The incidence of VTE in Asian patients with low BMI is considered to be lower than that in Western patients [11]. However, a recent study showed that the incidence of postoperative VTE after THA in Japanese patients was almost the same as in Western patients [12]. Second, surgeries were performed by three different surgeons. However, these surgeons were experienced senior hip surgeons who were familiar with each minimally invasive intermuscular approach. There was no significant difference in patient background characteristics, operative time, and intraoperative blood loss between the three approaches. Therefore, we believe that the impact of this factor may have been minor. Third, the same radiology specialist did not read the MDCT images and evaluate the presence of VTE. Fourth, this was a retrospective study, and the incidence of VTE after ambulation is unknown. Despite these limitations, we believe our data are worth presentation.

## Conclusions

Minimally invasive surgical techniques for total hip arthroplasty (THA) have become popular worldwide. However, venous thromboembolism (VTE) remains a major complication in patients, irrespective of the surgical approach. This study provides precise information based on multidetector-row computed tomography regarding the incidence of VTE in patients undergoing THA who were treated by the same protocol at a single institution. The results of these data indicate that the incidence of VTE is significantly higher in the anterolateral approach than in the anterolateral supine approach and direct anterior approach, especially on the non-operated side.
